# Supplementary material for: The VGNC: expanding standardized vertebrate gene nomenclature
Source: Genome Biol. 2023 May 12;24:115. doi: 10.1186/s13059-023-02957-2 (PMC10176861; doi:10.1186/s13059-023-02957-2)
Supplement: Supplementary file 5 — Additional file 5. Review history. [file 13059_2023_2957_MOESM5_ESM.docx]

Review History

**First round of review**

**Reviewer 1**

The VGNC work is important, and the resource is described well in this clearly written manuscript. The manuscript provides a detailed description of the process for assigning names of genes, and incremental statistics on approved gene names across a number of species, and describes the reasons (and numbers) for not approving of human gene names in the other vertebrates. The authors have recently published a paper that describes the revised website for VGNC (Tweedie, et al., NAR, 2021), and so some of the novelty (such as the website redesign) is lost. I did not detect substantial novelty that makes the publication, especially in GB, of clear value to the reader.

Minor comments

- The authors have published a description of the new (in 2019) VGNC website design in 2021; at least the Tweedie paper should be cited in the passage on Gene groups as this new tool was specifically described there ( l. 303 ) and is thus not novel.

- The text describing results illustrated in Figure 4 are a bit unclear- the text seems to indicate manual review and annotation have resolved some of these gene naming issues (i.e., l. 263 "All of these have been manually reviewed and approved in at least one VGNC species"...) So are these still concerns, or is these showing what has been addressed/corrected? Or a mixture? Perhaps a pie chart showing the starting point and a separate one showing the current, improved statistics would be useful to show impact of the VGNC efforts.

- Figure 5: the text describing Figure 5 does not make it clear that this is a navigational aid, a first impression is that this is just a description of the hierarchy of gene family nomenclature; without reading the Legend to the figure does it make clear that the image is clickable and takes the user to specific resources.

**Reviewer 2**

This manuscript was to expand Standardized vertebrate gene nomenclature across 7 mammalian species. The pipeline and manual curation methods have been well-developed in the past. This on-going effort will provide a great resource for scientific community. The authors mentioned they have coordinated with other groups on standardize gene nomenclature in other species, such as mouse, chicken, zebrafish etc. Not sure why at least mouse gene nomenclature was not included in this mspt for a pair-comparison with human gene nomenclature as mouse has been widely used as an animal model to study human diseases.

The resolution on a couple of Figures are very low (Figure 3, 5) and need be improved.

**Reviewer 3**

This is an important stop towards the correct gene nomenclature across vertebrates.

I understand the paper is for a database type. But I found it a bit dry to read because it focuses on a lot of the process of correcting and approving the nomenclature. There are some examples are given, but they are very much human-centric. Can we see some walk-through examples that this great work improved the understanding of non-human genes, since it's about vertebrate species?

I happened to have tried the website listed by the authors and searched the gene "paep", and it only displayed this gene for pig and chimpanzee. Is there any reason that this gene was not shown for other species?

**Authors Response**

**Point by point response to reviewer comments:**

We would like to thank all of the reviewers for their helpful comments, which we have responded to below and addressed in the manuscript revisions. We greatly appreciate their input as we feel these revisions have enabled us to improve the manuscript.

*Reviewer #1:*

*The VGNC work is important, and the resource is described well in this clearly written manuscript. The manuscript provides a detailed description of the process for assigning names of genes, and incremental statistics on approved gene names across a number of species, and describes the reasons (and numbers) for not approving of human gene names in the other vertebrates. The authors have recently published a paper that describes the revised website for VGNC (Tweedie, et al., NAR, 2021), and so some of the novelty (such as the website redesign) is lost. I did not detect substantial novelty that makes the publication, especially in GB, of clear value to the reader.*

The reviewer notes that some aspects of the VGNC project have been briefly described in our regular NAR Database issue publications, which are written primarily as articles describing the updates made to our HGNC and VGNC resources. To date, however, there has not been a dedicated VGNC publication describing the project comprehensively. We are aiming for this publication to serve as the primary citable description of the VGNC, necessitating a mention of all its main features, even if these have been described elsewhere. We do however feel that there is considerable novelty in this manuscript - a majority of the text describes aspects of the VGNC project that have not previously been published anywhere, and features such as the website are described more briefly since these have been cited elsewhere.

Content of this manuscript that has not been published elsewhere includes:

- the description of our pipeline determining 1:1 orthology relationships
- the database structure and update pipelines
- details of our internal rules that determine whether 1:1 orthologs in vertebrates can be automatically assigned the human gene nomenclature including examples
- the procedures followed by curators when performing manual curation
- the analysis of which human genes did not have named orthologs in VGNC species and descriptions of the progress made in gene naming as a result of this analysis
- details of assigning nomenclature to pseudogenes and to coding orthologs of human unitary pseudogenes
- the benefits of manual curation extending beyond gene nomenclature
- current major challenges faced in the project

*Minor comments*

*- The authors have published a description of the new (in 2019) VGNC website design in 2021; at least the Tweedie paper should be cited in the passage on Gene groups as this new tool was specifically described there ( l. 303 ) and is thus not novel.*

We apologise for this oversight and have added this citation (line 335).

*- The text describing results illustrated in Figure 4 are a bit unclear- the text seems to indicate manual review and annotation have resolved some of these gene naming issues (i.e., l. 263 "All of these have been manually reviewed and approved in at least one VGNC species"...) So are these still concerns, or is these showing what has been addressed/corrected? Or a mixture? Perhaps a pie chart showing the starting point and a separate one showing the current, improved statistics would be useful to show impact of the VGNC efforts.*

The pie chart in Figure 4 shows the number of human genes that had not had VGNC orthologs approved by January 2022. As described in the text, some of these genes were quickly approved after initial investigation, however the majority will require in depth manual curation to review gene models, evolutionary history and nomenclature before they can be approved. We have added another pie chart to Figure 4 showing the relative proportions of human genes that did already have named VGNC orthologs, to make it clearer how much progress the VGNC project has made to date; we have added additional text to the main manuscript making it clearer which genes are yet to be approved (lines 251-252, 279-280, 284-285, 287-288), and also highlighted in the legend to Figure 4 that the full list of these genes (listed in Additional File 3) also contains an indication of whether they have since been approved in VGNC.

*- Figure 5: the text describing Figure 5 does not make it clear that this is a navigational aid, a first impression is that this is just a description of the hierarchy of gene family nomenclature; without reading the Legend to the figure does it make clear that the image is clickable and takes the user to specific resources.*

We appreciate this feedback, and have added annotations to this figure (now Figure 6 in the revised manuscript) to make it clearer that this is a navigational tool.

*Reviewer #2:*

*This manuscript was to expand Standardized vertebrate gene nomenclature across 7 mammalian species. The pipeline and manual curation methods have been well-developed in the past. This on-going effort will provide a great resource for scientific community. The authors mentioned they have coordinated with other groups on standardize gene nomenclature in other species, such as mouse, chicken, zebrafish etc. Not sure why at least mouse gene nomenclature was not included in this mspt for a pair-comparison with human gene nomenclature as mouse has been widely used as an animal model to study human diseases.*

We had not mentioned mouse gene nomenclature as this is not part of the VGNC project remit, and rather is the responsibility of the Mouse Genome Nomenclature Committee, part of the Mouse Genome Informatics database. However, in response to this suggestion we have now added a section comparing the nomenclature of mouse and human orthologous genes (lines 187-197) to illustrate the consistency of gene naming between MGNC and HGNC/VGNC. The full list of curated human-mouse orthologs with gene symbol comparisons has been added in the new Additional File 2. We have also added text to the manuscript briefly describing our work to harmonize gene nomenclature for olfactory receptors, which previously had completely different nomenclature systems in human, mouse and rat (lines 197-200).

*The resolution on a couple of Figures are very low (Figure 3, 5) and need be improved.*

We are sorry that the resolution appears low - this is very disappointing as we submitted high resolution PNGs for figures 3 and 5 (now Figure 6 in this revised manuscript) and hence suspect that this could be a processing issue with the file(s) provided for review. We trust that the journal will check the resolution of the final figures is adequate and notify us if improvements are required.

*Reviewer #3:*

*This is an important stop towards the correct gene nomenclature across vertebrates.*

*I understand the paper is for a database type. But I found it a bit dry to read because it focuses on a lot of the process of correcting and approving the nomenclature. There are some examples are given, but they are very much human-centric. Can we see some walk-through examples that this great work improved the understanding of non-human genes, since it's about vertebrate species?*

The key remit of the VGNC is to approve and correct gene nomenclature, hence this is what the bulk of this manuscript is describing. In an attempt to make it more interesting and address this reviewer’s concerns we have added some specific examples, as requested, which concern enzymes that are coding in some lineages but not in others, and for which there are some known biological consequences of this change across species (lines 320-331, and the newly added Figure 5).

We have also added some text to briefly describe how our olfactory receptor curation led to improvements in the gene annotation data in NCBI RefSeq for a number of VGNC species (lines 385-389).

*I happened to have tried the website listed by the authors and searched the gene "paep", and it only displayed this gene for pig and chimpanzee. Is there any reason that this gene was not shown for other species?*

As we have tried to describe in the manuscript, VGNC gene naming is an ongoing project. We investigated the reasons that *PAEP* has not been approved in all VGNC species to date, and the primary reason is that our orthology comparison pipeline fails to identify a clear 1:1 ortholog in all species. *PAEP* is a member of a complex gene family and the region contains different numbers of annotated genes in different species; therefore careful manual curation is required to determine the orthology relationships between species and link the *PAEP* gene symbol to the correct gene models. This work will be completed in due course.

**Second round of review**

**Reviewer 1**

Thanks for the clarifications and additions, no further comments.
